# Follistatin impacts Tumor Angiogenesis and Outcome in Thymic Epithelial Tumors

**DOI:** 10.1038/s41598-019-53671-8

**Published:** 2019-11-22

**Authors:** Stefan Janik, Christine Bekos, Philipp Hacker, Thomas Raunegger, Ana-Iris Schiefer, Leonhard Müllauer, Cecilia Veraar, Balazs Dome, Walter Klepetko, Hendrik Jan Ankersmit, Bernhard Moser

**Affiliations:** 1 Christian Doppler Laboratory for Diagnosis and Regeneration of Cardiac and Thoracic Diseases, Medical University Vienna, Vienna, Austria; 2 Department of Otorhinolaryngology, Head and Neck Surgery, Medical University Vienna, Vienna, Austria; 3 Department of Obstetrics and Gynecology, Division of General Gynecology and Gynecologic Oncology, Medical University Vienna, Vienna, Austria; 4 Division of Thoracic Surgery, Department of Surgery, Medical University Vienna, Vienna, Austria; 5 Clinical Institute of Pathology, Medical University Vienna, Vienna, Austria; 6 Department of Anaesthesiology, General Intensive Care and Pain Medicine, Medical University of Vienna, Vienna, Austria; 7 Division of Thoracic Surgery, Department of Surgery, Comprehensive Cancer Centre Vienna, Medical University Vienna, Vienna, Austria; 8 Head FFG Project “APOSEC“, FOLAB Surgery, Medical University Vienna, Vienna, Austria

**Keywords:** Outcomes research, Surgical oncology

## Abstract

Tumor angiogenesis is a key factor in the progression of thymic epithelial tumors (TETs). Activin A, a member of the TGFβ family, and its antagonist Follistatin are involved in several human malignancies and angiogenesis. We investigated Activin A and Follistatin in serum and tumor tissue of patients with TETs in relation to microvessel density (MVD), WHO histology classification, tumor stage and outcome. Membranous Activin A expression was detected in all tumor tissues of TETs, while Follistatin staining was found in tumor nuclei and cytoplasm. Patients with TETs presented with significantly higher Activin A and Follistatin serum concentrations compared to healthy volunteers, respectively. Follistatin serum concentrations correlated significantly with tumor stage and decreased to physiologic values after complete tumor resection. Follistatin serum concentrations correlated further with MVD and were associated with significantly worse freedom from recurrence (FFR). Low numbers of immature tumor vessels represented even an independent worse prognostic factor for FFR at multivariable analysis. To conclude, the Activin A - Follistatin axis is involved in the pathogenesis of TETs. Further study of Follistatin and Activin A in TETs is warranted as the molecules may serve as targets to inhibit tumor angiogenesis and tumor progression.

## Introduction

Thymic epithelial tumors (TETs), comprising thymomas and thymic carcinomas (TCs) are extremely rare malignancies with an annual incidence of only 3.2 cases per million^1^. However, though rare, TETs are the most common malignancies in the anterior mediastinum in adults.

Clinical behavior and outcome differs significantly between thymomas and TCs. Thymomas, accounting for 72% to 89% of TETs, are characterized by a more benign tumor behavior compared to TCs and display a unique association with the paraneoplastic autoimmune disease Myasthenia Gravis (MG)^2–5^. Indeed, more than 30% of thymoma patients have thymoma associated MG (TAMG), while 10% to 20% of myasthenic patients have thymomas^6^. Conversely, TCs represent a minor group of TETs that are not associated with paraneoplastic MG, which show a more aggressive tumor behavior with worse outcome and more recurrences compared to thymomas^7,8^. The Masaoka-Koga staging system, which differentiates TETs according to level of invasiveness into four stages, ranging from non-invasive (stage I) to metastasized TETs (stage IV), is most commonly used for staging of TETs^9^. Recently a new TNM staging system for TETs was introduced^10^.

Surgical tumor resection represents the mainstay of therapy even in case of recurrent disease or in TETs with pleural metastases^11^. Recurrences can occur even decades after radical tumor resection, and therefore life-long oncologic follow up is recommended^2,12,13^. In our previous works we could demonstrate that different systemic inflammatory proteins, such as CRP or Fibrinogen and indices formed from inflammatory cell counts, such as neutrophil-to-lymphocyte ratio (NLR) were associated with higher tumor stage and worse prognosis^3,14^. However, despite improved insights also into genetic profiles of TETs, there exist no established markers for diagnosis or to predict tumor recurrence during oncologic follow-up of TETs^15–17^. Subsequently, a better insight into the pathogenesis of TETs and identification of new biomarkers are still needed.

Activin A, a member of the TGFβ superfamily of cytokines, binds to type II activin receptors on the cell surface, which leads to recruitment and phosphorylation of type I activin receptors that results in intracellular phosphorylation and activation of Smad proteins. The latter finally ends up in translocation of the activated Smad proteins into the nucleus and regulation of gene transcription^18,19^. Follistatin represents a natural antagonist of Activin A that prevents interaction of Activin A with type I and II activin receptors, resulting in endocytosis of the Activin A/Follistatin complex and lysosomal degradation^19^.

Within the past years, a deregulation or mutation of the activin signaling axis was found in numerous malignancies. Follistatin has also tumorigenic and proangiogenic functions, at least partially through antagonizing Activin A^20,21^.

Therefore, we hypothesized that Activin A and Follistatin may play a role in the pathophysiology of TETs. It was the primary aim of the study to evaluate whether serum Activin A and Follistatin serum concentrations correspond to histologic tumor subtype, stage and tumor behavior. In addition, we investigated whether TETs express Activin A or Follistatin, and whether there is an association between Activin A and Follistatin with tumor microvessel density (MVD) and outcome.

## Results

### Activin A and follistatin serum concentrations in thymic epithelial tumors

**Figure 1:**
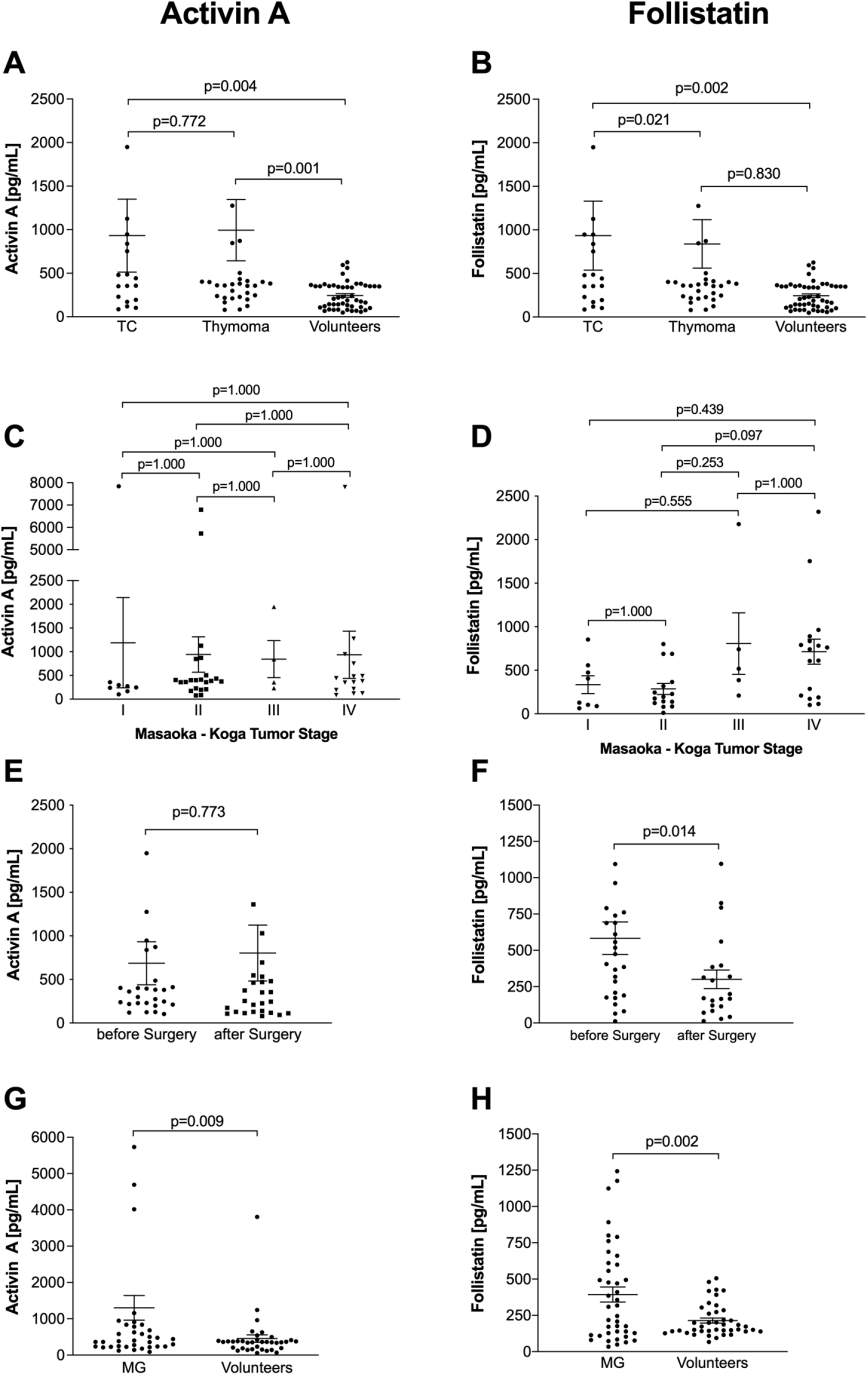
Activin A and Follistatin serum concentrations in patients with thymic epithelial tumors and Myasthenia Gravis Patients without TETs. Plots show preoperative Activin A and Follistatin serum concentrations in patients with thymic carcinomas (TCs), thymomas and healthy volunteers (**A**,**B**), and in patients with TETs according to Masaoka - Koga tumor stage (**C**,**D**). Further, the impact of tumor resection on postoperative Activin A and Follistatin serum levels is indicated (**E**,**F**). In addition, analysis of Activin A and Follistatin serum concentrations are illustrated in myasthenic patients (MG) compared to volunteers (**G,H**). Scatter dot plots are indicated as mean ± standard error of the mean.

Activin A and Follistatin serum concentrations were significantly elevated in patients with TETs compared to healthy volunteers (p = 0.009 and p = 0.002; respectively). Patients with TCs and thymomas had significantly higher Activin A serum concentrations compared to volunteers (p = 0.004 and p = 0.001; Fig. 1A). Conversely, we detected highest Follistatin serum concentrations in patients with TCs, which were significantly higher compared to thymomas (p = 0.021) and volunteers (p = 0.002; Fig. 1B) (Table 1).

**Table 1:** Activin A and Follistatin serum concentrations in patients with thymic epithelial tumors.

Variables	n	Activin A	p-value	Follistatin	p-value
		mean (median) ± SD (SEM)		mean (median) ± SD (SEM)	
**TETs**					
TC	15	933.1 (399.4) ± 1771.4 (418.9)		756.4 (609.4) ± 669.0 (157.7)	
Thymoma	31	995.1 (360.8) ± 1961.5 (352.3)		352.1 (208.7) ± 293.9 (56.6)	
Volunteers	49	244.8 (207.8) ± 152.8 (21.8)	<0.001^a^	254.4 (187.3) ± 164.5 (24.5)	0.010^a^
**WHO Classification**					
MNT	2	222.8 (222.8) ± 195.1 (138.0)		93.8 (93.8) ± 115.4 (81.6)	
A	4	2181.0 (341.7) ± 3774.8 (1887.4)		483.9 (473.9) ± 364.8 (210.6)	
AB	8	435.0 (377.5) ± 281.5 (99.5)		328.7 (268.9) ± 252.3 (103.0)	
B1	5	254.3 (272.9) ± 114.4 (46.7)		106.0 (108.8) ± 36.1 (18.0)	
B2	7	1964.4 (453.1) ± 2685.7 (949.5)		277.9 (245.4) ± 198.8 (75.1)	
B3	5	319.6 (358.1) ± 89.1 (51.4)		705.2 (800.1) ± 280.3 (125.4)	
TC	15	933.1 (399.4) ± 1961.5 (352.3)	0.425^b^	756.4 (609.4) ± 669.0 (157.7)	0.080^b^
**Masaoka – Koga Tumor Stage**					
I	8	1190.5 (260.3) ± 2688.6 (950.6)		334.3 (267.2) ± 286.1 (101.1)	
II	19	942.2 (390.9) ± 1747.4 (372.5)		285.9 (194.5) ± 246.0 (63.5)	
III	5	843.2 (597.0) ± 782.2 (391.1)		806.5 (517.8) ± 791.2 (353.9)	
IV	17	934.7 (381.2) ± 1929.4 (498.2)	0.988^b^	713.4 (741.1) ± 589.1 (142.9)	0.037^b^
**Longitudinal Analysis**					
before surgery	30	685.5 (360.8) ± 1287.4 (247.8)		505.9 (376.2) ± 564.9 (103.1)	
after surgery	30	802.8 (253.0) ± 1664.7 (320.4)	0.773^c^	210.4 (177.3) ± 280.4 (51.2)	0.014^c^
**Paraneoplastic MG**					
TAMG	17	1091.1 (382.9) ± 1966.2 (476.9)		395.5 (281.8) ± 292.3 (68.9)	
non TAMG	29	909.3 (358.7) ± 1857.4 (328.3)	0.751^d^	592.7 (406.1) ± 612.3 (117.8)	0.211^d^
**Myasthenia Gravis**					
MG pos.	47	1209.9 (437.1) ± 1915.7 (279.4)		393.6 (308.9) ± 331.5 (51.8)	
Volunteers*	47	421.8 (353.1) ± 547.5 (79.9)	0.009^e^	214.6 (172.7) ± 113.4(17.7)	0.002^d^

**Abbreviations:** *TETs, thymic epithelial tumors; TC, thymic carcinoma; WHO, World Health Organization classification of histologic tumor subtype; MNT, micronodular thymoma; MG, myasthenia gravis; TAMG, thymoma associated myasthenia gravis; n, number of patients.*

Preoperative Activin A (pg/mL) and Follistatin (pg/mL) serum values are shown in patients with thymic epithelial tumors with regards to tumor subtype, WHO classification, Masaoka – Koga tumor stage and paraneoplastic thymoma-associated Myasthenia Gravis (TAMG). We performed also longitudinal analysis of Activin A and Follistatin serum concentrations before and after tumor resection. *In addition, we evaluated serum levels of myasthenic patients without thymic malignancy compared to sex- (p = 0.834) and age-matched (p = 0.079) volunteers. ^a^Kruskal-Wallis test; ^b^One-way ANOVA; ^c^paired samples *t*-test; ^d^Unpaired Student’s *t*-test; ^e^Mann-Whitney-U test.

### Serum follistatin and Masaoka – Koga tumor stage

Preoperative Follistatin but not Activin A serum concentration was significantly different according to Masaoka - Koga tumor stage (p = 0.037 and p = 0.988; respectively; Fig. 1C,D). In particular, Follistatin serum concentrations were 806.5 ± 791.2 pg/mL and 713.4 ± 589.1 pg/mL in stage III and IV TETs, which was significantly different compared to 334.3 ± 286.1 pg/mL and 285.9 ± 246.0 pg/mL in stage I and II TETs (p = 0.005) (Table 1).

### Decreased follistatin serum concentrations after complete tumor resection

Preoperative Follistatin serum concentrations decreased significantly after complete tumor resection (505.9 ± 564.9 pg/mL vs. 210.4 ± 280.4 pg/mL; p = 0.014; Fig. 1F). After radical tumor resection postoperative Follistatin serum concentrations reached physiologic serum levels, similar to those found in healthy volunteers (210.4 ± 280.4 pg/mL vs. 254.4 ± 164.5 pg/mL; p = 0.465). With regard to Activin A serum concentrations, tumor resection did not significantly influence Activin A serum levels (p = 0.773; Fig. 1E) (Table 1).

### Impact of myasthenia gravis on Activin A and follistatin serum concentrations

Activin A and Follistatin serum concentrations did not significantly differ in patients with TAMG compared to TETs without MG (p = 0.751 and p = 0.211; respectively; Table 1). In addition, we analyzed serum samples of MG patients without thymic malignancy compared to sex- (p = 0.834) and age-matched (p = 0.079) healthy volunteers. Myasthenic patients showed also significantly elevated Activin A (p = 0.009; Fig. 1G) and Follistatin (p = 0.002; Fig. 1H) serum concentrations compared to controls (Table 1). Furthermore, we differentiated myasthenic patients based on their thymic pathology into patients with TETs, those with true thymic hyperplasia and those with follicular thymic hyperplasia. However, benign thymic pathology did not significantly impact Activin A and Follistatin serum concentration in patients with MG (p = 0.804 and p = 0.722; respectively).

### Follistatin und Activin A expression in thymic epithelial tumors

**Figure 2:**
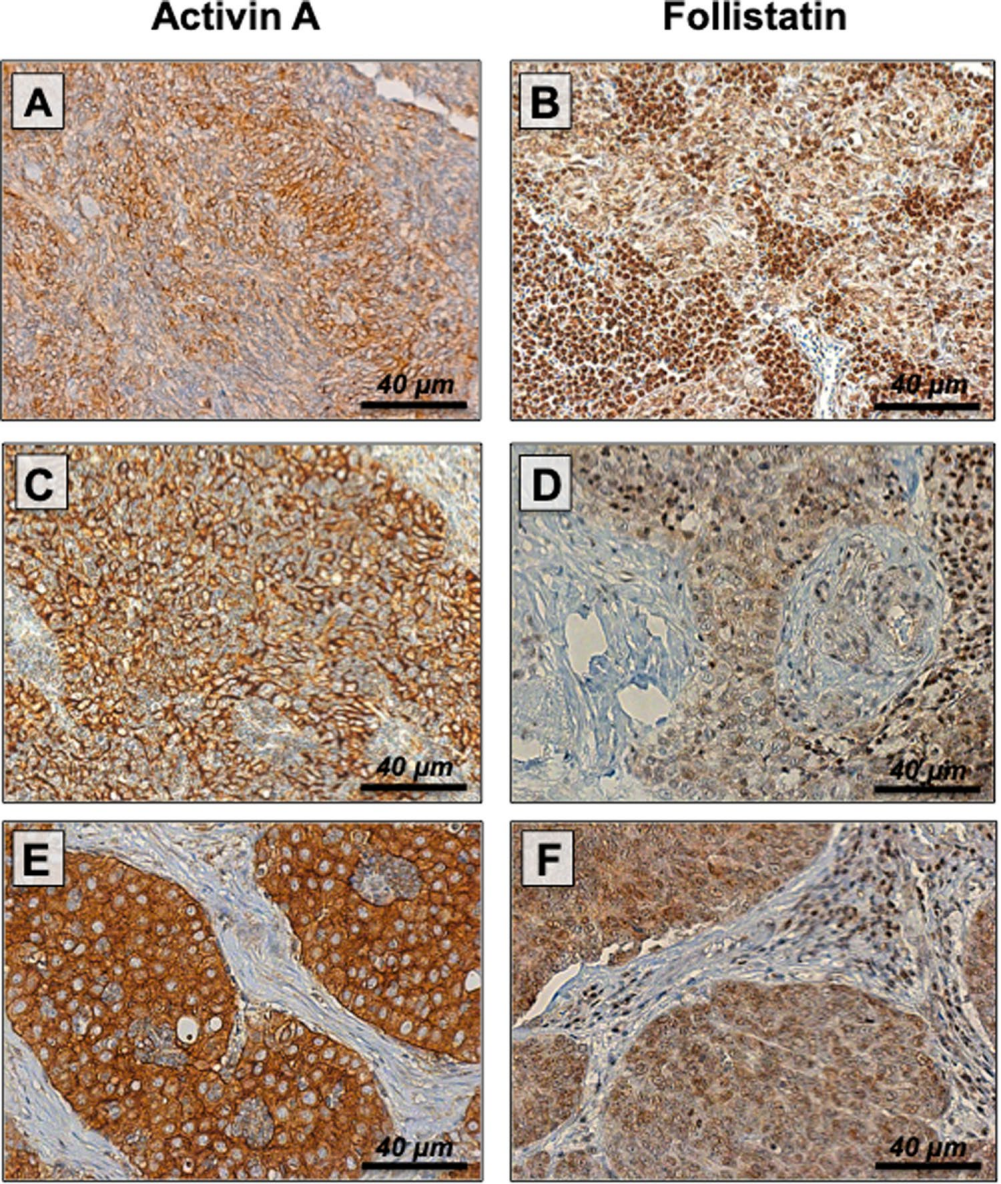
Activin A and Follistatin expression in thymic malignancies. Strong cytoplasmic and membranous Activin A expression was found in all TETs. Representative Activin A staining is shown in a WHO type AB (**A**), B2 (**C**), and B3 (**E**) thymoma. Conversely, nuclear and cytoplasmic Follistatin staining was found in MNT (**B**), B2 thymoma (**D**), and TC (**F**) (magnification × 400; scale bar: 40 μm). *WHO*, *World Health Organization*; *TC*, *thymic carcinoma*; *MNT*, *micronodular thymoma*.

We performed Follistatin and Activin A staining in 95 specimens of TETs (4 micronodular thymomas (MNT), 14 A, 14 AB, 10 B1, 21 B2, 17 B3 thymomas, 15 TCs; Fig. 2). Membranous Activin A expression was detected in all TETs without significant differences between thymomas and TCs or according to WHO subtype. Conversely, Follistatin staining was found in tumor nuclei and cytoplasm (p = 1.000). There was a trend towards absent nuclear Follistatin expression in TCs compared to thymomas. In particular, 75% of all TCs displayed absent Follistatin in nuclei, whereas absent nuclear Follistatin expression was found in 19% of all thymomas (p = 0.053). There were no semiquantitative differences in Follistatin staining intensity between thymomas and TCs.

### Follistatin serum levels correspond with microvessel density

**Figure 3:**
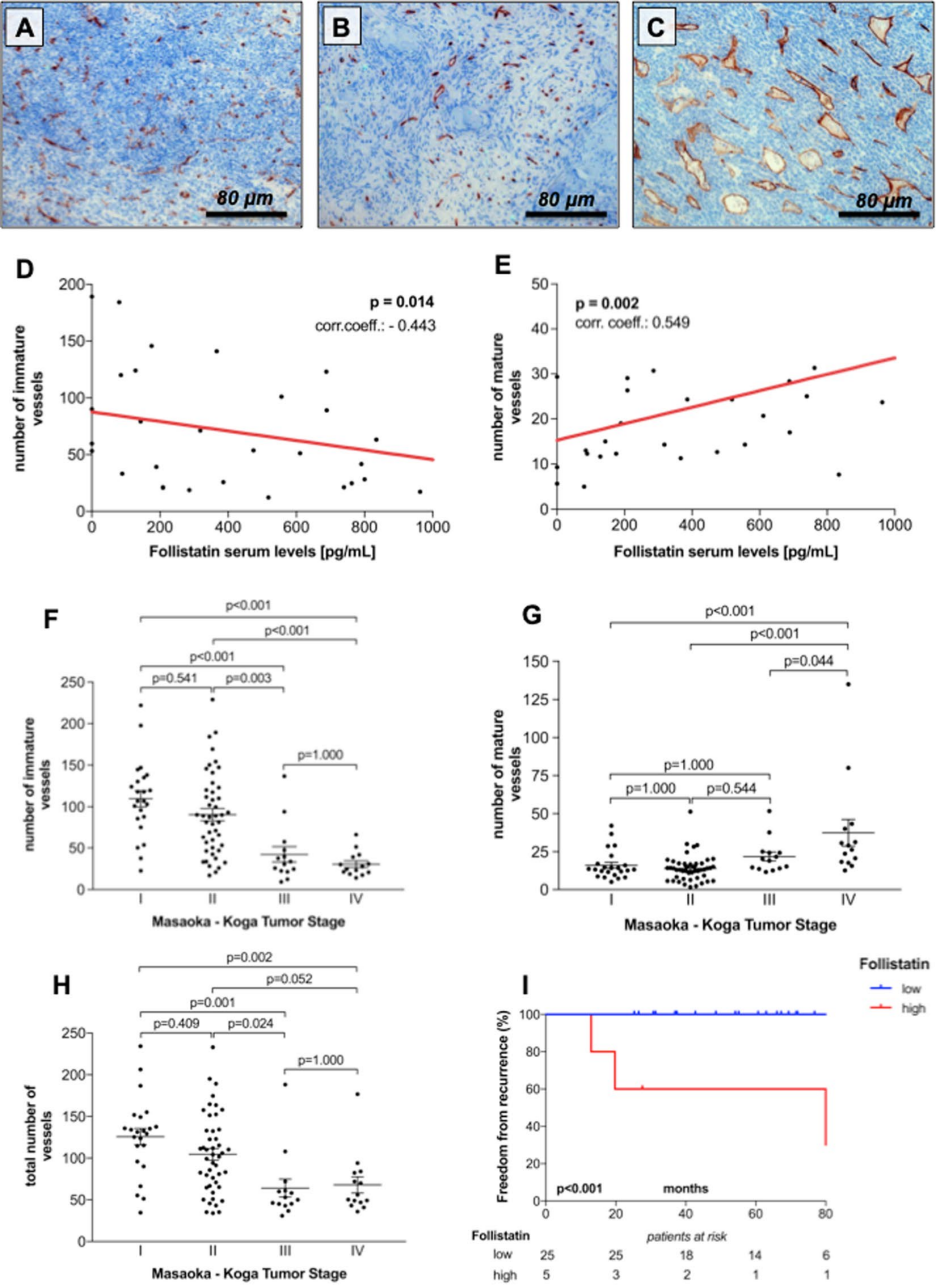
Microvessel Density in thymic epithelial tumors. CD34 staining of a Masaoka - Koga stage I WHO type B2 thymoma (**A**), a stage II A thymoma (**B**) and a stage III A thymoma (**C**) are shown (200 × magnification; scale bar: 80 μm). Preoperative Follistatin serum concentrations showed a significant negative correlation (**D**) with the number of immature tumor vessels (r: −0.443; p = 0.014), and a significantly positive correlation (**E**) with the amount of mature tumor vessels (r: 0.549; p = 0.002). While the amount of immature tumor vessels decreased with higher tumor stage (**F**), the number of mature tumor vessels increased with level of invasiveness (**G**). Similarly, total numbers of tumor vessels decreased from stage I to stage IV tumors (**H**). High preoperative Follistatin serum levels were associated with significantly worse FFR (**I**).

We performed Spearman correlation analyses to evaluate whether serum Follistatin impacts MVD and vascular architecture in TETs (Fig. 3A–C). Follistatin serum levels showed a significant negative correlation with the number of immature (r: −0.443; p = 0.014) tumor vessels (Fig. 3D). In addition, there was also a significantly positive correlation between Follistatin and mature tumor vessels (r: 0.549; p = 0.002 Fig. 3E). As expected, immature tumor vessels correlated significantly with mature vessels (r: −0.440; p < 0.001) and total number of vessels (r: 0.932; p < 0.001), respectively.

Furthermore, we explored the possibility that other systemic inflammatory markers, such as Fibrinogen, CRP, Heat Shock Proteins or molecules of the RAGE axis, may also correlate with specific vascular architecture of TETs. However, beside serum Follistatin none of the abovementioned proteins showed significant correlation with mature, immature or total tumor vessels (Table 2). Nonetheless we found a significant correlation between Follistatin and CRP (r: 0.576; p = 0.006) and Follistatin with Fibrinogen (r: 0.512; p = 0.018).

**Table 2:** Spearman correlation was performed to analyze relationships between microvessel density and different systemic inflammatory markers.

Variables	Immature Vessels	Mature Vessels	Total Nr. of Vessels
Follistatin *pg/mL*	−0.443*	0.549*	−0.272
Activin A *pg/mL*	0.347	−0.228	0.339
HSP27 *pg/mL*	−0.228	0.200	−0.140
HSP70 *ng/mL*	0.067	0.158	0.163
sRAGE *pg/mL*	−0.083	0.072	−0.072
esRAGE *pg/mL*	−0.154	−0.119	−0.240
HMGB1 *ng/mL*	0.246	−0.086	0.226
CRP *mg/dL*	−0.196	0.166	−0.175
Fibrinogen *mg/dL*	−0.351	0.234	−0.298
Immature Vessels *number*	1	−0.440*	0.932*
Mature Vessels *number*	−0.440*	1	−0.188
Total Nr. of Vessels *number*	0.932*	−0.188	1

**Abbreviations:** HSP, heat shock proteins; sRAGE, soluble receptor for advanced glycation end products; esRAGE, elevated endogenous secretory RAGE; HMGB1, high-mobility group box 1 protein; CRP, C-reactive protein.

Follistatin serum concentrations correlated significantly with the absolute number of immature tumor vessels (r: −0.443; p = 0.014). There was also a strong significant correlation with Follistatin and absolute number of mature tumor vessels (r: 0.549; p = 0.002). As expected, the number of immature tumor vessels correlated significantly with the number of mature vessels (r: −0.440; p < 0.001) and total number of vessels (r: 0.932; p < 0.001). *p-value < 0.05.

### Lowest number of immature, but highest number of mature vessels in thymic carcinomas

The mean number of immature and mature vessels was 79.5 ± 51.6 and 19.0 ± 16.8, respectively. TCs showed significantly higher numbers of mature (29.8 ± 17.1 vs. 17.2 ± 16.5), and lower numbers of immature tumor vessels (25.1 ± 10.8 vs. 90.0 ± 50.5) compared to thymomas (p = 0.010; p < 0.001). With regards to WHO thymoma subtypes, the highest number of immature vessels was found in MNT 121.1 ± 31.6 and WHO type AB thymomas 115.4 ± 25.8, respectively.

Moreover, TCs showed significantly lower immature to mature (1.11 ± 0.89 vs. 9.26 ± 10.6; p < 0.001) and immature to total vessel ratios (0.47 ± 0.16 vs. 0.81 ± 0.16; p < 0.001) compared to thymomas. Regarding to thymoma subtypes, WHO type B1 thymomas showed the highest immature to mature vessel ratios (15.2 ± 15.3), and AB thymomas the highest ratio of immature to total number of vessels of 0.89 ± 0.05, respectively. It is noteworthy that we detected the highest ratio of mature to total amount of tumor vessels in TCs, which was significantly higher compared to thymomas (0.53 ± 0.16 vs. 0.19 ± 0.16; p < 0.001) (Table 3).

**Table 3:** Microvessel Density in TETs of patients in regard to histology, tumor stage and myasthenia gravis.

Variables	Nr.	Absolute Number of Vessels			Tumor Vessel Ratios		
		*mean ± SEM*			*mean ± SEM*		
		Immature	Mature	Total	Imm./Mat.	Imm./Tot.	Mat./Tot.
**WHO Classification**							
MNT	4	121.1 ± 15.8	15.8 ± 1.9	136.8 ± 14.9	8.2 ± 1.6	0.88 ± 0.03	0.12 ± 0.03
A	14	114.5 ± 16.0	19.0 ± 3.6	133.4 ± 15.8	9.8 ± 2.6	0.83 ± 0.03	0.17 ± 0.03
AB	14	115.4 ± 6.9	13.7 ± 1.7	129.0 ± 6.5	10.8 ± 2.0	0.89 ± 0.01	0.11 ± 0.01
B1	10	87.1 ± 13.4	10.0 ± 1.8	97.0 ± 12.4	15.2 ± 4.8	0.86 ± 0.04	0.14 ± 0.04
B2	21	82.8 ± 11.6	13.5 ± 1.5	96.2 ± 11.3	10.1 ± 2.9	0.82 ± 0.03	0.18 ± 0.03
B3	17	52.3 ± 9.5	27.7 ± 7.3	80.0 ± 11.7	3.3 ± 0.8	0.64 ± 0.05	0.36 ± 0.05
TC	14	25.1 ± 2.9	29.8 ± 4.8	54.9 ± 4.7	1.1 ± 0.2	0.47 ± 0.04	0.53 ± 0.03
*p* - value^a^		<0.001	0.008	<0.001	0.005	<0.001	<0.001
**Masaoka – Koga Tumor Stage**							
I	23	109.6 ± 9.7	16.1 ± 2.0	125.7 ± 9.9	8.8 ± 1.2	0.86 ± 0.02	0.14 ± 0.02
II	45	91.6 ± 7.3	13.9 ± 1.3	105.5 ± 6.9	11.4 ± 1.9	0.83 ± 0.02	0.17 ± 0.02
III	13	41.1 ± 9.2	22.4 ± 3.0	63.5 ± 10.8	2.1 ± 0.5	0.60 ± 0.05	0.40 ± 0.05
IV	13	28.9 ± 3.9	38.7 ± 9.7	67.6 ± 9.6	1.1 ± 0.3	0.47 ± 0.05	0.53 ± 0.05
*p* - value^a^		<0.001	<0.001	<0.001	0.001	<0.001	<0.001
**Myasthenia Gravis**							
positive	29	77.6 ± 6.5	22.8 ± 9.3	106.6 ± 9.3	7.2 ± 1.5	0.76 ± 0.04	0.24 ± 0.04
negative	68	83.8 ± 9.0	17.4 ± 1.4	95.0 ± 6.0	8.2 ± 1.3	0.75 ± 0.02	0.25 ± 0.02
*p* - value^b^		0.594	0.272	0.295	0.664	0.803	0.803

**Abbreviations:** MNT, Micronodular thymoma; TC, thymic carcinoma; Nr., number of patients; SEM, standard error of the mean.

Absolute number and ratios of tumor vessels are indicated with respect to histologic tumor subtype, tumor stage and paraneoplastic MG. Imm./Mat. Ratio was calculated by dividing the relative number of immature vessels by the relative number of mature vessels, while Imm./Tot. Ratio and Mat./Tot. Ratio were calculated by dividing the relative number of immature and mature tumor vessels by the total number of all tumor vessels, respectively. ^a^One-way ANOVA; ^b^Unpaired Student’s *t*-test.

### Masaoka–Koga tumor stage and microvessel density

MVD and vascular architecture showed significant differences according to Masaoka – Koga tumor stage. The highest number of mature tumor vessels (38.7 ± 9.7) was detected in stage IV TETs (p < 0.001; Fig. 3G), while the number of immature vessels decreased gradually from stage I TETs (109.6 ± 46.4) to stage IV TETs (28.9 ± 3.9; p < 0.001; Fig. 3F). Similar, highest mature to total vessel ratio of 0.53 ± 0.05 and lowest immature to total vessel ratio of 0.47 ± 0.05 was found in metastasized stage IV TETs (Table 3).

### Impact of microvessel density and serum parameters on outcome

Next, we performed Kaplan-Meier survival analysis to evaluate how Follistatin and Activin A serum concentrations and MVD impact freedom from recurrence (FFR) and cause specific survival (CSS). We calculated Youden-Indices to find optimal cut-off values for Activin A and Follistatin for predicting tumor recurrence. The highest Youden-Indices of 0.231 and 0.742 were found at cut-off values of 483.3 pg/mL and 776.5 pg/mL for Activin A and Follistatin, respectively. With respect to MVD, median number of mature (14.3), immature (71.3) and total tumor vessels (91.3) was used for dichotomizing patients into lower and higher subgroups, respectively. Low numbers of immature and total vessels were associated with significantly worse FFR (p = 0.001; p = 0.006) and CSS (p = 0.014; p = 0.017), respectively. High preoperative Follistatin serum concentrations were associated with significantly worse FFR (p < 0.001; Fig. 3I), but not CSS (p = 1.000), while Activin A serum levels did not impact outcome (Table 4).

**Table 4:** Survival Analyses.

Variables	Freedom From Recurrence				Cause Specific Survival			
	1y	5y	10y	*p*^*a*^	1 y	5 y	10 y	*p*^*a*^
**Activin A**								
Low	100	100	100		100	100	100	
High	100	85.7	85.7	0.083	100	100	100	1.000
**Follistatin**								
Low	100	100	100		100	100	100	
High	100	60.0	30.0	<0.001	100	100	100	1.000
**Mature Vessels**								
Low	95.5	93.2	93.2		97.7	95.3	91.8	
High	100	88.2	88.2	0.548	100	97.8	93.8	0.636
**Immature Vessels**								
Low	95.7	80.7	74.0		97.7	92.8	84.1	
High	100	100	100	0.001	100	100	100	0.014
**Total Number of Vessels**								
Low	95.6	81.2	81.2		97.8	93.0	84.7	
High	100	100	95.8	0.006	100	100	100	0.017

Youden - Index was used to find optimal cut-off values of 483.3 pg/mL and 776.5 pg/mL for preoperative Activin A and Follistatin serum concentrations, respectively. Conversely, we used the median for mature, immature and total number of vessels for dichotomizing patients into low and high subgroups. 1-year, 5-year and 10-year freedom from recurrence and cause specific survival rates are indicated. ^a^Log-rank test.

### Univariable and multivariable Cox-Regression analyses

Finally we performed univariable and multivariable Cox-regression analyses to assess the prognostic power of following clinical characteristics on FFR and CSS: histology (TC vs. thymoma), Masaoka-Koga tumor stage (III-IV vs. I-II), MG (neg. vs. pos.), sex (female vs. male), number of immature (low vs. high) and mature (high vs. low) tumor vessels and total number of vessels (low. vs. high).

At univariable analysis, presence of TC (HR 6.10; p = 0.002), tumor stage III-IV (HR 6.10; p = 0.003), low number of immature (HR 14.8; p = 0.010) and low number of total tumor vessels (HR 6.48; p = 0.016) represented significantly worse prognostic factors for FFR. Among these variables, only low number of immature tumor vessels stayed an independent worse prognostic factor at multivariable analysis. In particular, low number of immature tumor vessels showed a 35-fold higher risk for tumor recurrence compared to TETs with high number of immature tumor vessels (HR 35.3; p = 0.021). With respect to CSS, only presence of TC (HR 15.2; p = 0.002) was a significant worse prognostic factor at univariable testing (Table 5).

**Table 5:** Univariable and Multivariable Cox-Regression Analyses.

	Univariable Analysis			Multivariable Analysis		
	HR	p-value	95% CI	HR	p-value	95% CI
**Freedom From Recurrence**						
Histology (TC vs. Thymoma)	6.10	0.002	1.89–19.61	2.08	0.317	1.99–8.40
Tumor Stage (III-IV vs. I-II)	6.10	0.003	1.85–20.0	1.86	0.392	0.45–7.75
Myasthenia Gravis (neg. vs. pos.)	1.19	0.761	0.27–2.62			
Sex (female vs. male)	0.76	0.623	0.26–2.27			
Immature Vessels (low vs. high)	14.8	0.010	1.90–114.8	35.3	0.021	1.70–734.1
Mature Vessels (high vs. low)	1.42	0.550	0.45–4.52			
Total Nr. of Vessels (low vs. high)	6.48	0.016	1.41–29.8	0.24	0.220	0.02–2.35
Activin A (high vs. low)	33.3	0.599	0.0–100.0			
Follistatin (high vs. low)	100.0	0.510	0.0–100.0			
**Cause Specific Survival**						
Histology (TC vs. Thymoma)	15.2	0.002	2.73–83.3	3.36	0.178	0.58–19.61
Tumor Stage (III-IV vs. I-II)	500	0.212	0.36–100.0	1.00	0.930	0–100.0
Myasthenia Gravis (neg. vs. pos.)	2.11	0.496	0.25–18.1			
Sex (female vs. male)	0.36	0.233	0.07–1.94			
Immature Vessels (low vs. high)	81.2	0.235	0.06–100.0			
Mature Vessels (high vs. low)	0.65	0.639	0.11–3.91			
Total Nr. of Vessels (low vs. high)	75.6	0.241	0.06–100.0			
Activin A (high vs. low)*	—	—	—			
Follistatin (high vs. low)*	—	—	—			

**Abbreviations:** HR, Hazard Ratio; CI, Confidence Interval; Nr, Number.

Prognostic impact of histology, tumor stage, Myasthenia Gravis, sex, number of immature and mature vessels, and total number of tumor vessels on freedom-from-recurrence and cause-specific survival are shown. The following cut-off values were used for dichotomizing patients into high and low subgroups: Immature Vessels: 71.3; Mature Vessels: 14.3; Total Number of Vessels: 91.3; Activin A: 483.3 pg/mL; Follistatin: 776.5 pg/mL. *Univariable cox-regression analyses of Activin A and Follistatin serum levels for predicting cause specific survival were not possible due to lack of events.

## Discussion

Herein we investigated for the first time the role of Activin A and Follistatin serum concentrations and tumor expression in TETs. We found significantly elevated Activin A and Follistatin serum concentrations in patients with TETs compared to healthy volunteers. The findings were in line with the literature reporting of elevated Activin A serum levels in patients with malignant pleura mesothelioma^22^, lung adenocarcinoma^23^, breast cancer and prostate cancer^24^, hepatocellular carcinoma^25^, and endometrial and cervical carcinoma^26^. In our study, preoperative Activin A serum levels did not significantly correlate with Masaoka –Koga tumor stage, WHO classification or outcome, which was in contrast to literature, where increased Activin A serum levels were commonly associated with higher tumor stage and poor outcome.

Conversely to Activin A, elevated Follistatin serum concentrations were described only in few malignancies, including ovarian cancer^27^, lung adenocarcinoma^28^, hepatocellular carcinoma^29^, and prostate cancer^30^. In particular, high Follistatin serum levels were associated with shorter overall survival (OS) and poor prognosis in hepatocellular carcinoma^29^, emergence of bone metastases in prostate cancer^30^ or tumor proliferation of lung adenocarcinoma cells^28^. This is in agreement with our data, showing that highest Follistatin serum concentrations were found in patients with advanced stage TETs and TCs, and that high preoperative serum levels were associated with significantly worse FFR.

It is commonly assumed that the cancerogenic function of Follistatin depends primarily on its role as antagonist of Activin A^21^. Follistatin could prevent Activin A induced growth inhibition and apoptosis and therefore Follistatin fosters cell proliferation and tumor growth. Stove C. *et al*.^31^ found that *in-vitro* melanoma cells express activin receptors and that treatment with Activin A led to growth inhibition and apoptosis, which could be counteracted by addition of Follistatin^31^. In accordance to that, Chen F. *et al*.^28^ demonstrated that cultured lung adenocarcinoma cells secreted Follistatin, and that inhibition of Follistatin led to significantly augmented Activin A induced apoptosis^28^.

In addition, Follistatin also has effects on tumor-associated angiogenesis, which mostly depends on the inhibition of Activin A. Endothelial cells constitutively express activin receptors and VEGF, and binding of Activin A leads to inhibition of proliferation of cultured endothelial cells by downregulation of p21 and downregulation of VEGF expression^19,32^. In accordance to that, inhibition of p21 increased endothelial cell proliferation and resistance to Activin A mediated growth inhibition^33^. Moreover, Follistatin induces also angiogenesis by binding and activating Angiogenin, a potent activator of angiogenesis^34^.

Within our study, we could further link for the first time the pro-angiogenic function of serum Follistatin to MVD in TETs. Follistatin serum levels showed a significant negative correlation with absolute number of immature tumor vessels, and a significant positive correlation with mature tumor vessels. Herein, we defined maturity of tumor vessels as presence of vessel lumen that depends on the proliferation of endothelial cells. Therefore, the amount and characteristic pattern of tumor vessels in TETs most likely depend on proliferation of endothelial cells, which might be influenced by Follistatin.

Tumor angiogenesis represents one of the hallmarks of cancer and is essential for tumorigenesis, tumor growth and metastasis^35^. Already in 2002, Tomita *et al*. showed that mean MVD of TETs significantly correlated with tumor stage and invasiveness^36^. Reica *et al*. (2010) classified tumor vessels in thymomas as mature, intermediate and immature, and they further suggested that tumor invasiveness might correspond with tumor vessel subtype^37^. Recently, Pfister F. *et al*.^38^ could show that vascular architecture and expression profile of key angiogenic growth factors differ significantly in TETs. In particular, densest vascular networks of predominantly small vessels were found in A thymomas, while TCs showed the least dense vascular network with almost large capillaries and highest VEGF expression^38^.

Similar to that, patients with TCs showed the lowest MVD, but the highest amount of mature tumor vessels in our cohort. Furthermore, we could demonstrate that the number of immature vessels gradually decreased with level of invasiveness, while the amount of mature tumor vessels gradually increased with tumor stage. Indeed low numbers of immature tumor vessels represent a significantly worse prognostic factor for FFR with a 35-fold increased risk for recurrent disease. We managed to link the pro-angiogenic function of Follistatin with MVD and further to demonstrate that tumor vessel subtypes differ significantly according to WHO classification. Hence, manipulation of the Activin A and Follistatin system in TETs may represent a potential target to interfere with tumor angiogenesis, tumor growth and metastases in TETs.

According to data from the Unigene database, Follistatin is not expressed in normal (physiologic) thymus^21^. Herein, we could demonstrate that Follistatin and Activin A were generally expressed in TETs. We found constant membranous Activin A staining in all TETs, which was most likey caused by the usage of an anti-human Anti-Activin A receptor type IB antibody. Conversely, Follistatin as a soluble antagonist of Activin A, was detected in cytoplasm and nucleus of tumor cells. Soluble Follistatin molecules envelop Activin A and thus block the access of Activin A to type I and type II receptor binding sites^39^. Furthermore, Follistatin and Activin A binding complexes can interact with cell-surface proteoglycans followed by internalization and lysosomal degradation^40^, which could be an explanation of Follistatin expression in the cytoplasm of TETs. With respect to the nuclear function of Follistatin, Lin C. *et al*.^41^ (2016) could demonstrate that nuclear Follistatin prevented cultured human lung epithelial cells and mouse lunge tissue from reactive oxygen induced apoptosis and that down-regulation of Follistatin promoted apoptosis^41^. Similar, in HeLa cells, nuclear expression of Follistatin delayed glucose deprivation-induced apoptosis by attenuating RNA synthesis, which represents a key process of cellular energy homeostasis and cell survival^42^. Altogether we assume that nuclear expression of Follistatin in malignant thymic epithelial cells might cause cell-survival and inhibition of apoptosis, while the cytoplasmic Follistatin expression might result from degradation of Follistatin – Activin A complexes.

Follistatin serum concentrations decreased significantly after radical tumor resection, indicating that Follistatin serum levels are at least partially related to the presence of malignant thymic epithelial cells. Whether Follistatin is secreted directly by tumor cells, as demonstrated for human lung adenocarcinoma cells^28^, or indirectly by immune cells in response to tumor cells or as acute phase reaction^43^, remains elusive. Therefore, although our data are interesting, further studies are necessary to clarify the molecular and cellular effects of Activin A and Follistatin in TETs.

Our study has some limitations due to the mostly retrospective study design, the single center experience and the low number of patients, which limit our ability to draw conclusions. Moreover, although our data are interesting, we could not provide experimental data of Follistatin and Activin A in TET cell lines to further confirm our results. However, the strength of the study is that we could link the pro-angiogenic function of Follistatin to MVD in TETs, which corresponds to Masaoka-Koga tumor stage and outcome.

Taken together, we could demonstrate that preoperative Activin A and Follistatin serum concentrations were significantly elevated in patients with TETs and MG compared to controls. Follistatin serum levels were highest in patients with TCs and advanced tumor stage. Most importantly, Follistatin correlated significantly to MVD, which in turn was significantly different according to WHO subtype and Masaoka – Koga tumor stage. Number of immature tumor vessels represented a significant prognostic factor for FFR. Hence, manipulation of the Activin A and Follistatin system in TETs may represent a potential target to interfere with tumor angiogenesis and tumor progression in TETs.

## Material and Methods

### Study population

This study was conducted at the Department of Thoracic Surgery, Medical University of Vienna. From this cohort, patients with the diagnosis of a primary TET, who underwent surgical tumor resection, were included. Between 1999 and 2014, 98 patients with thymic malignancy were identified to meet criteria. Three patients with thymic neuroendocrine tumors (TNETs) were excluded.

Clinical and sociodemographic characteristics for each patient were obtained from medical hospital records, surgical and pathological reports, and imaging findings. Within our cohort, we had 56 female (58.9%) and 39 male (41.1%) patients with a mean age of 57.2 ± 15.9 years.

WHO classification was used for differentiating TETs into thymomas and TCs^44^. Accordingly, thymomas were classified as WHO type A, AB, B1, B2, B3 thymomas, and micronodular thymomas (MNT)^44,45^. We had 4 MNT (4.2%), 14 type A (14.7%), 14 AB (14.7%), 10 B1 (10.5%), 21 B2 (22.1%), 17 B3 thymomas (17.9%) and 15 TCs (15.8%; squamous cell carcinomas, SCCs), respectively. Within our cohort, we had 8 mixed type thymomas, including 7 B2/B3 and 1 B1/B2 thymomas. For the purpose of statistical analysis of this manuscript, WHO mixed type thymomas were classified according to the more malignant part (e.g. B2/B3 thymoma was classified as B3 thymoma).

Masaoka – Koga staging was as follows: stage I 23 (24.2%), stage II 45 (47.4%), stage III 13 (13.7%) and stage IV 14 cases (14.6%). Twenty - nine (30.5%) out of these 95 TETs were associated with TAMG.

### Outcome analysis

The mean and median follow-up time was 95.5 and 72.4 months (range: 0.1–525.1 months), respectively. Within oncologic follow-up, periodic chest CT-scans were performed postoperatively every 3 to 6 months for the first three years followed by annual CT-scans. Thirteen (13.5%) patients experienced recurrent disease (local: n = 2; regional: n = 5; distant: n = 6) and six patients died from TETs (6.3%). According to the recommendations of the International Thymic Malignancy Interest Group (ITMIG), we used CSS and FFR as main oncologic outcome parameters within this study^12^. CSS was calculated in all patients and was defined as time from date of surgery to date of death from a TET, while unrelated deaths or unknown causes of death were censored. In contrast, FFR was only calculated in patients after radical tumor resection (R0) and was defined as time from date of surgery to date of recurrence.

### Therapy

Among those 95 TETs who underwent surgical tumor resection, completeness of surgical resection could be achieved in 83 cases (87.4%), while R1 and R2 status were obtained in 9 (9.4%) and 3 (3.2%) of TETs, respectively. Forty-six patients (48.4%) underwent only surgical tumor resection, while the remaining 49 patients (51.6%) received multimodal therapy. In particular, 11 patients (11.6%) underwent neoadjuvant chemotherapy (ChT; n = 5) or concomitant radiochemotherapy (RChT; n = 6), while 42 patients (44.2%) received adjuvant therapy, including adjuvant ChT (n = 3), RT (n = 31) and RChT (n = 8), respectively.

### Preparation of specimens and immunohistochemistry

In our clinical institute of pathology, tumor sections are processed from the capsule, every 1–2 cm of tumor tissue and from tumor regions, which appear different on gross inspection. However, our immunohistochemical analyses were done on selected slides from one block per patient that represented the main tumor component with a very homogenous histology.

Formaldehyde-fixed and paraffin embedded human specimens of all patients (n = 95) with TETs were available for immunohistochemical stainings. Staining was performed by using the automated Ventana Benchmark® platform (Ventana Medical Systems, Tucson, AZ, USA) according to standard protocol of the Clinical Institute of Pathology^46^. We used monoclonal rabbit anti-human Anti-Activin A Receptor Type IB antibody (ab204655, Abcam, Cambridge, UK) and monoclonal mouse anti-human Follistatin antibody IgG_2a_ (MAB669, R&D Systems, Minneapolis, MN, USA) as primary antibodies to assess Activin A and Follistatin expression in TETs. In addition, we used monoclonal mouse anti-human CD34 antibody (LEICA, NovoCastra, Clone QBEnd/10, Nussloch, Germany) to assess microvascular density.

### Evaluation of immunoreactivity in thymic epithelial tumors

We used a qualitative score to evaluate staining intensity of Activin A and Follistatin in tumor cells. Staining intensities were calculated as 0 (none), weak (1), moderate (2) or strong (3). Specimens were only counted as positive if more than 90% of tumor cells showed strong (3) Activin A or Follistatin expression.

### Microvessel density

As already published and described by Weidner *et al*. 1992, we assessed microvessel density (MVD) using the “hotspot” method^47^. Briefly, slides were screened at low magnification to identify the areas with the greatest number of CD34 stained vessels (“hotspot”). MVD was determined by counting all CD34 positive vessels at 200x magnification (corresponding to 0.95 mm²) in 95 TETs. One specimen was removed due to lack of evaluable remaining thymic tissue. Three representative hotspots were calculated for each specimen.

Tumor vessels were further differentiated into immature and mature vessels according to the classification of Gee MG *et al*. based on the presence of perfused lumen and perivascular cells^48^. Accordingly, immature vessels are differentiated from intermediate and mature vessels by the presence or absence of vessel lumen, whereas intermediate and mature vessels are characterized by the presence or absence of perivascular cells. Herein, we used a simplified method and differentiated only between immature and mature vessels. Within the Results section, total number of immature, mature and all tumor vessels are indicated as well as following ratios: Ratio 1 (immature/mature), Ratio 2 (immature/total) and Ratio 3 (mature/total).

### Enzyme-linked immunosorbent assays (ELISA)

Preoperative serum samples were available of 46 patients with TETs (17 TAMG, 29 non-TAMG) and 30 patients with MG for analysis of Activin A and Follistatin blood levels. Forty-nine sex and age-matched healthy volunteers were used as controls. Pre - and postoperative serum samples were available in a subset of 30 patients with TETs. Postoperative serum samples were collected in patients who underwent primary surgery 6 to 12 months ago, who did not receive adjuvant therapy within the last month, and who did not have signs of recurrence or a 2^nd^ malignancy.

To assess Activin A and Follistatin serum concentrations, we used the commercial available human Activin A ELISA kit (R&D Systems, Minneapolis, MN, USA, DuoSet® human Activin A, DY338) and human Follistatin ELISA kit (R&D Systems, Minneapolis, MN, USA, DuoSet® human Follistatin, DY669). All tests were performed according to the Manufacture’s protocols. Samples were measured in duplicates and researches performing the assays were blinded to the groups associated with each sample.

Additionally to Activin A and Follistatin serum concentrations, Fibrinogen, CRP, heat shock protein 27 and 70, and molecules of the RAGE axis (sRAGE, esRAGE, HMGB1) as previously shown, were correlated with MVD^3,14,46,49^.

### Statistical methods

Statistical analysis of data was performed using SPSS software (version 21; IBM SPSS Inc., IL, USA). The type of test used is indicated in the table and/or the result section. All data are reported as mean ± standard deviation (SD) within result section. Chi-square test was used to investigate the association between nominal variables. Unpaired Student’s *t-*test and one-way ANOVA were used to compare means of two or more than two independent groups with normal (Gaussian) distributions, respectively. Post-hoc Tukey’s - B and Bonferroni correction were used in case of multiple testing. Mann-Whitney-U test and Krusky-Wallis test were performed to analyze non-normal distributed variables with two or more than two groups, respectively. Paired Student’s *t*-test was performed for analyzing means of two dependent groups. Pearson correlation (r) was done to analyze linear relationships between two numerical measurements, while Spearman correlation analysis was applied for analysis of ranked variables. Kaplan-Meier analyses and Log-rank test were assessed for survival analysis. Uni - and multivariable Cox-regression analysis was used to evaluate the prognostic impact of different clinical variables on CSS and FFR. The receiver operating characteristic (ROC) curve and Youden-Index were calculated to find optimal cut-off values of 483.3 pg/mL for Activin A and 776.6 pg/mL for Follistatin for predicting tumor recurrence.

### Ethics statement

Ethical approval was obtained from the Ethics Committee of the Medical University of Vienna (302/2011). All participating patients and control subjects gave their written informed consent, and all experiments were performed in accordance with the approved ethical guidelines.
